# Internal and marginal discrepancies of monolithic zirconia, polymer infiltrated ceramic, and lithium disilicate crowns: an *in vivo* study

**DOI:** 10.1590/0103-644020256664

**Published:** 2025-12-01

**Authors:** Larissa Mendonça de Miranda, Sophia Queiroz Marques dos Santos, Anne Heloyse Teixeira Crispim, Isabelle Helena Gurgel de Carvalho, Amanda Maria de Oliveira Dal Piva, Yu Zhang, Rodrigo Othávio de Assunção e Souza

**Affiliations:** 1 Department of Dentistry, Federal University of Rio Grande do Norte, Natal, RN, Brazil.; 2 University of Amsterdam, Department of Dental Materials Science. Amsterdam. Netherlands; 3 University of Pennsylvania, Department of Preventive and Restorative Sciences. United States.

**Keywords:** CAD/CAM, Ultratranslucent zirconia, Marginal discrepancy, Internal discrepancy, Replica technique

## Abstract

Evaluate the marginal and internal discrepancies of Lithium Disilicate (LD), Monolithic Zirconia (Zr), and Polymer-Infiltrated Ceramics (PIC) crowns in vivo. Thirty patients requiring posterior single crowns were divided into three groups (n = 10 each): LD (IPS e.max CAD), Zr (Zircon Fit Plus), and PIC (VITA Enamic). After tooth preparation and temporary crown placement, impressions were taken for the fabrication of the crown. Marginal (MD) and Internal (ID) Discrepancies were measured before cementation using the replica technique and optical stereomicroscopy. ID was assessed by measuring the light body silicone thickness in occlusal, cusp, axial, and chamfer regions, while MD was evaluated at the chamfer finish line. ID was analyzed by two-way ANOVA and Tukey tests, while MD was analyzed by one-way ANOVA (α=5%). For ID, ANOVA revealed that the factors “material”, “region”, and their interaction were significant (p < 0.05). Although ID values varied by material and region, all remained within clinically acceptable limits. When comparing the experimental groups, PICo (226.0±84.7A μm) and PICc (165.6±71.9AB μm) exhibited the highest ID values, while LDax (87.9±17.3D μm) and PICax (91.4±36.2D μm) had the lowest. For MD, ANOVA revealed no significant differences between the materials (p = 0.4287): Zr (108.4 ± 34.6 μm), LD (95.7 ± 13.6 μm), and PIC (114.5 ± 42 μm). ID and MD were comparable among the evaluated crown materials, with all values falling within clinically acceptable limits. However, the lack of reported acceptable clinical thresholds and limited sample size highlight the need for larger-scale studies and long-term follow-ups to assess the longevity of restorative treatments.

## Resumo

Avaliar as discrepâncias marginais e internas de coroas Dissilicato de Lítio (LD), Zircônia Monolítica (Zr) e Cerâmicas Infiltradas por Polímero (PIC) in vivo. Trinta pacientes necessitando de coroas unitárias posteriores foram divididos em três grupos (n=10 cada): LD (IPS e.max CAD™), Zr (Zircon Fit Plus) e PIC (VITA Enamic). Após o preparo dental e colocação das coroas temporárias, foram feitas impressões para a confecção das coroas. As discrepâncias marginais (MD) e internas (ID) foram medidas antes da cimentação utilizando a técnica de réplica e estereomicroscopia óptica. A ID foi avaliada pela medição da espessura de silicone de corpo leve nas regiões oclusal, de cúspide, axial e de chanfro, enquanto a MD foi avaliada na linha de acabamento de chanfro. A ID foi analisada por ANOVA de dois fatores e testes de Tukey, enquanto a MD foi analisada por ANOVA de um fator (α=5%). Para a ID, a ANOVA mostrou que os fatores “material”, “região” e sua interação foram significativos (p<0,05). Embora os valores de ID variem de acordo com o material e a região, todos permaneceram dentro dos limites clinicamente aceitáveis. Ao comparar os grupos experimentais, PICo (226,0±84,7^A^ μm) e PICc (165,6±71,9^AB^ μm) apresentaram os maiores valores de ID, enquanto LDax (87,9±17,3^D^ μm) e PICax (91,4±36,2^D^ μm) tiveram os menores. Para a MD, a ANOVA indicou que não houve diferenças significativas entre os materiais (p=0,4287): Zr (108,4 ± 34,6 μm), LD (95,7 ± 13,6 μm) e PIC (114,5 ± 42 μm). A ID e a MD foram comparáveis entre os materiais de coroas avaliados, com todos os valores dentro dos limites clinicamente aceitáveis. No entanto, a falta de limiares clínicos aceitáveis relatados e o tamanho amostral limitado destacam a necessidade de estudos em maior escala e de acompanhamentos a longo prazo para avaliar a longevidade dos tratamentos restauradores.

**Figure:**
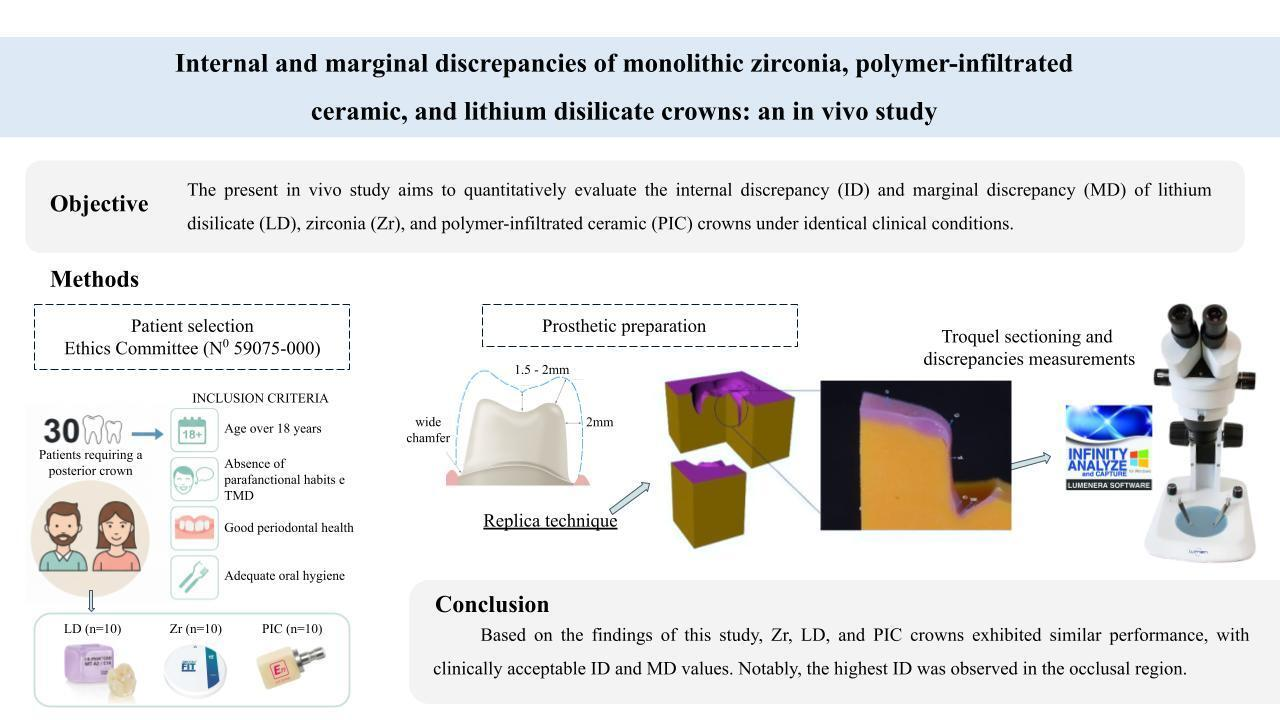

## Introduction

Dental ceramics have been widely used due to their favorable mechanical properties and excellent optical characteristics, allowing for aesthetic reproduction of enamel and dentin. ^1^ Various types of dental ceramic blocks are available for CAD/CAM (Computer-Aided Design/Computer-Aided Manufacturing) fabrication, such as lithium disilicate (LD), indicated for the fabrication of onlays, anterior and posterior crowns, veneers, and anterior fixed prostheses of up to three units. ^2^ LD consists of a glass matrix and filler particles, providing outstanding aesthetic properties due to its translucency, in addition to a flexural strength of 350 MPa.^3^ Clinical studies report that for posterior monolithic single crowns, LD has a survival rate of up to 96.5% after 10 years of follow-up. ^4^ Despite its longevity, failures are often associated with caries, loss of retention, and ceramic fractures and have been reported mainly in the posterior regions due to the higher masticatory loads. ^5^

To minimize fractures in ceramic restorations, monolithic zirconia (Zr) crowns have been used as an alternative to LD due to their superior mechanical strength. ^6^ Several generations of zirconia are available on the dental market. The first generation (3Y-TZP) is characterized by high strength and opacity, making it suitable only for infrastructures, abutments, and implants. ^7^ The second generation (high-translucent-HT) features improved translucency, allowing for its use in monolithic restorations without the need for veneering ceramics, while maintaining high mechanical strength (>850 MPa). ^1^ This increase in translucency is attributed to a larger zirconia grain size and a reduced amount of aluminum oxide sintering aids. The grain size increases because, with the reduction of Al₂O₃ as a sintering aid, second-generation zirconia is sintered at 1500°C - 1580°C, resulting in larger grains compared to first-generation zirconia, which is typically sintered at 1450°C. ^8^ However, its translucency remains significantly lower than that of glass ceramics used for aesthetic restorations. To address this limitation, the third generation, known as ultratranslucent (UT) zirconia, was developed. This material achieves high translucency through a reduced Al₂O₃ content, an increased yttrium oxide concentration (4 mol.% - 6 mol.%), and an increase in the proportion of the cubic phase to up to 80%. ^8^ Although UT zirconia exhibits lower fracture strength (400 - 700 MPa) than previous generations, it still offers superior mechanical properties compared to glass ceramics. ^6^ UT zirconia is indicated for monolithic restorations, including endocrowns, tooth- and implant-supported posterior and anterior crowns, veneers, and anterior fixed prostheses of up to three units. ^9^

In the pursuit of esthetic restorative materials with an elastic modulus comparable to dentin ^10^ while offering easier milling and clinical adjustment than glass-matrix or polycrystalline ceramics and allowing intraoral repair, resin-matrix ceramics were developed. ^11^ According to the classification proposed by Gracis et al. (2015), 11 these materials are categorized into three groups based on their inorganic content: resin nanoceramic, glass ceramic in a resin interpenetrating matrix, and zirconia-silica ceramic in a resin interpenetrating matrix. Resin nanoceramic consists of a highly polymerized resin matrix reinforced with ~80 wt% nanoceramic particles, represented by Lava Ultimate (3M ESPE). ^11^. On the other hand, the glass ceramic in a resin interpenetrating matrix comprises a feldspathic ceramic network (86%) infiltrated with polymers (14% - TEGDMA and UDMA),^12^ with an elastic modulus of 30-32 GPa, lower than that of fully ceramic materials, represented by VITA Enamic (VITA Zahnfabrik). Manufacturers often describe these as hybrid ceramics, given the interconnected polymer network that reduces brittleness and increases stress tolerance. ^13^. Finally, the zirconia-silica ceramic in a resin interpenetrating matrix shows variability in organic matrix composition and ceramic content. ^11^ ^)^ Under masticatory forces, these materials undergo elastic and plastic deformation that dissipates energy, reducing the risk of catastrophic failure and enhancing resistance to crack initiation and propagation. ^14^ Clinically, they do not require sintering after milling, enabling immediate cementation. ^15^ ^)^ Resin-matrix ceramics are indicated for multiple applications, including full-coverage anterior and posterior crowns, inlays, onlays, and implant-supported crowns. ^16^ Reported survival rates reach 84.8% for inlays and 82.4% for overlays after three years of clinical service. ^12^

The scientific literature presents divergent results regarding the marginal and internal discrepancy of crowns fabricated with LD, PIC, and Zr. ^17^ According to the literature, vertical marginal discrepancy (MD) refers to the vertical misfit between the internal surface of the restoration and the outer edge of the finish line. Internal discrepancy (ID) refers to the distance between the internal surface of the restoration and the occlusal or axial wall of the preparation, representing the thickness of the cement layer. ^18^ Ideally, restorations should fit as precisely as possible, since high MD values lead to greater exposure of the cement in the oral environment, promoting its dissolution. ^17^ This, in turn, facilitates biofilm accumulation, which can cause inflammation and damage to periodontal tissues. ^19^ Meanwhile, high ID values negatively affect the mechanical stability of the restoration, reducing retention and fracture resistance. ^20^ Studies indicate that LD presents clinically acceptable marginal and internal adaptation, depending on factors such as fabrication technique and heat treatment.^21^ ^,^ ^22^ PIC, due to not requiring post-milling sintering, tends to present less dimensional distortion, resulting in more accurate adaptation.^23^ Although UT zirconia offers excellent mechanical strength, it may show greater discrepancies due to the sintering shrinkage inherent to its manufacturing process.^24^

Few in vivo studies have directly compared the marginal and internal adaptation of LD, Zr, and PIC crowns using standardized methodologies. This lack of comparative clinical data limits the ability to make evidence-based decisions regarding material selection. Therefore, the present in vivo study aims to address this gap by quantitatively evaluating the ID and MD of these materials under identical clinical conditions. The tested hypotheses were that: a) Monolithic UT zirconia and PIC crowns exhibit similar MD and ID to lithium disilicate crowns, and that b) The measured crown region influences internal discrepancy.

## Materials and methods

**Figure 1:**
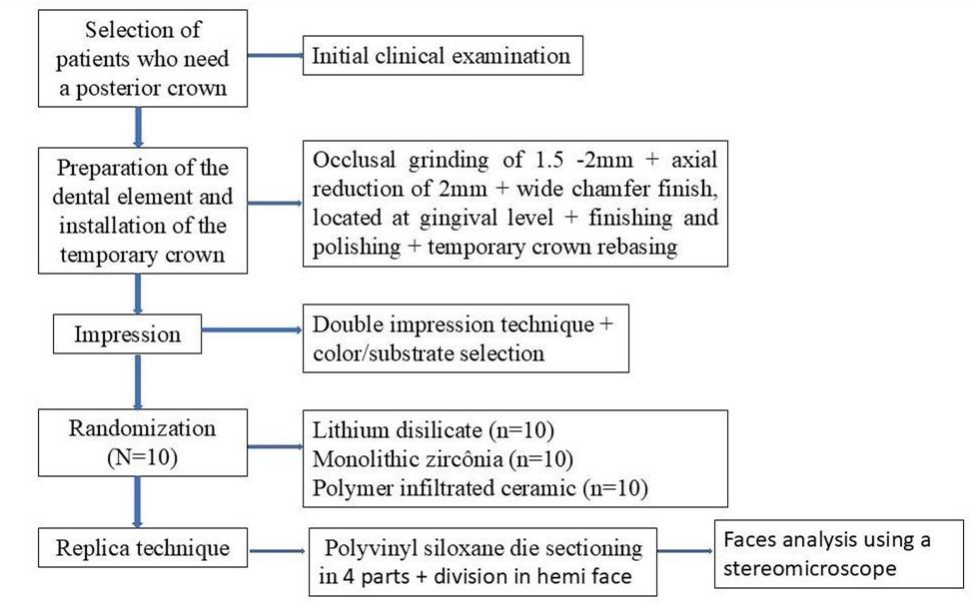
Flowchart of the study's design.

The trademark, manufacturers, and chemical compositions of the materials that were used in this study are listed in Table 1. The study design flowchart is illustrated in Figure 1.

**Table 1:** Materials used in the study for the crowns' manufacturing and the replica technique.

Trademark	Material Type	Composition	Manufacturer
IPS e.max CAD	Lithium disilicate	SiO_2_ ,Li_2_O, K_2_O, MgO, Al_2_O_3_, P_2_O_5_ and other oxides.	Ivoclar
Prettau 4 Previous Dispersive	Crystalline ceramic ultratranslucent zirconia	ZrO_2_ + HfO_2_ +Y_2_O_3_: ≥ 99.0% Y_2_O_3_: ≤ 6% HfO_2_: ≤ 5% Al_2_O_3_: ≤ 0.5% Other oxides: ≤ 0.5%	Zirkonzahn
VITA Enamic	Polymer-infiltrated ceramic	Feldspar inorganic ceramics (86%), urethane dimethacrylate, tri-ethylene glycol dimethacrylate (14%) (SiO2, Al2O3, Na2O, K2O, B2O3, CaO, TiO2, TEGDMA, UDMA)	VITA Zahnfabrik
Express XT - Dense Folder	Addition silicone	Al (35%) + Cristobalite (30%)+ Vinyl- polydimethylsiloxane (20%)+ Hydrocarbons (5%)+Amorphous silica (<5%)+ Polysiloxane dimethyl copolymer (<5%) Quartz (<5%)	3M ESPE

### Patient selection

After receiving Ethics Committee approval (N^0^ 59075-000), thirty ^30^ patients requiring at least one posterior full coverage crown (premolars and molars) were included in this study. Inclusion criteria were patients older than 18 years; absence of parafunctional habits and temporomandibular disorders (TMD); good periodontal health (absence of gingivitis and generalized periodontitis); and adequate oral hygiene. Patients with severe periodontal diseases, users of removable prostheses, and patients without stable occlusion, with multiple missing teeth and without unilateral or bilateral posterior support, were excluded. Before starting treatment, all patients were required to sign the Informed Consent Form (ICF), which allowed them to participate in this research.

### Study protocol

Patients were randomized into three groups, using a simple randomization process, according to the restorative material: lithium disilicate (LD - IPS e.max CAD, Ivoclar Vivadent, Schaan, Liechtenstein) (*n* = 10), zirconia (Zr - Zircon Fit Plus-Talmax, Curitiba, Paraná) (*n* = 10), or polymer infiltrated ceramics (PIC - VITA Enamic, VITA Zahnfabrik, Bad Sackingen, Germany). All stages were performed in a standardized and systematized manner for all groups. At the first visit, the patients underwent anamnesis, and then the impressions of both upper and lower arches with an irreversible hydrocolloid impression material (Jeltrate, Dentsply, Mount Waverley, Australia) were taken, followed by the preparation of plaster models (Gypsum type III). The models were sent to the dental laboratory for the manufacturing of CAD/CAM temporary restorations. The dental remnant was evaluated for the need for periodontal surgery with crown increase, and indication of being reconstructed with fiberglass post and filling core or metal post and core.

### Prosthetic preparation

The tooth was prepared with 1.5 to 2 mm occlusal reduction and axial wall reduction of 2 mm, from 8° to 10°, using diamond tips (#4138, #2135, and #3195, KG Sorensen) with a wide chamfer ending located at the gingival level. Finishing and polishing were performed with fine diamond burs (#4138F), multilaminate tungsten carbide drill, and Arkansas polisher (Komet, SP, Brazil) using a multiplier counter-angle (inLab SW v.22, Dentsply Sirona, Gmbh, Germany). The final substrate of the preparation could be dentin, fiberglass post, composite resin, or cast metallic core; however, the finishing region should be performed in the tooth area. Although some teeth had undergone previous endodontic treatment, all preparations were standardized, followed by finishing and polishing, and all preparation margins were placed in dentin, minimizing the influence of substrate heterogeneity on the fit of the restorations. After tooth preparation, the temporary crowns were rebased with acrylic resin in the corresponding color (Dencôr lay, Dental Articles- Clássico SP, Brazil), adjusted, polished, and cemented with temporary cement (Provicol, Voco).

After one week, molds were obtained with the double impression technique using addition silicone (Express XT, 3M ESPE, St Paul, MN, United States) and retraction cords nº000 and nº00 (Ultradent, Indaiatuba/SP/Brazil) for gingival retraction. The color selected by the same operator using the VITA Classical scale (Vita Zhanfabrick). Then, the molds were sent to the same laboratory for the preparation of the working models and final crowns according to the material selected. The allocation of the sample (teeth undergoing rehabilitation) into groups was performed randomly through a simple draw, conducted separately for each tooth. For this purpose, the group names were equally distributed into black opaque envelopes. After the clinical step of impression-taking of the preparations, the patient randomly selected one envelope for each compromised tooth undergoing rehabilitation.

In the laboratory, the mold was poured, and a stone model was generated, which was then scanned (Scanner Zirkonzan). Modellier Zirkonzahn modeling software (In Lab SW Sirona) was used for modeling on the preparation, with minimum thickness definitions of 0.3 - 0.5 mm and cementation space 0.04 - 0.05 mm in all faces. The design was sent to the Programill PM7 milling machine (Ivoclar Vivadent), where Yellow 2.50, 1.00, and 0.50 cutters were used. Then, the LD and Zr crowns were sintered following the manufacturer's instructions.

Zirconia was sintered at a temperature rise of 10°C/min to a final temperature of 1530°C with a dwell time of 2 hours, followed by natural cooling. For DL, the initial working temperature was 403°C. The temperature was then increased at a rate of 60°C/min until it reached 770°, which was maintained for 10 s. The temperature was then increased at 30°C/min until it reached 850°C, where it was held for 10 minutes before beginning to decrease and cool down slowly.

### Replica technique

Prior to cementation, the temporary crowns were removed, and the preparation was cleaned with a soft brush (Microtuf, DHpro, São Paulo, SP, Brazil) and a mixture of pumice paste and water for 20 s on each face, followed by cleaning with air and water jets.

After checking the adaptation and contact points inside the mouth, a replica technique was performed to evaluate MD and ID. For this, before cementation, each crown was filled with a low-consistency addition silicone fluid (Express XT, Light Body, 3M ESPE, St Paul, MN, United States) and kept in position on the respective preparation inside the mouth, with digital pressure of approximately 50N, for 5 min, until the silicone completely polymerized. This step was carried out by the same previously calibrated evaluator, so that the force performed is similar. After, the crown was removed with the adhered light body silicone. Next, a small portion of putty silicone (Express XT, 3M ESPE, St Paul, MN, United States) was manipulated and inserted inside the crown, in contact with the fluid silicone. After 5 min, the silicone troquel formed by both silicones was separated from the crown. The cementation line, represented by the light body silicone, was measured to evaluate the ID and MD discrepancies in each crown.

### Troquel sectioning and discrepancies measurements

**Figure 2:**
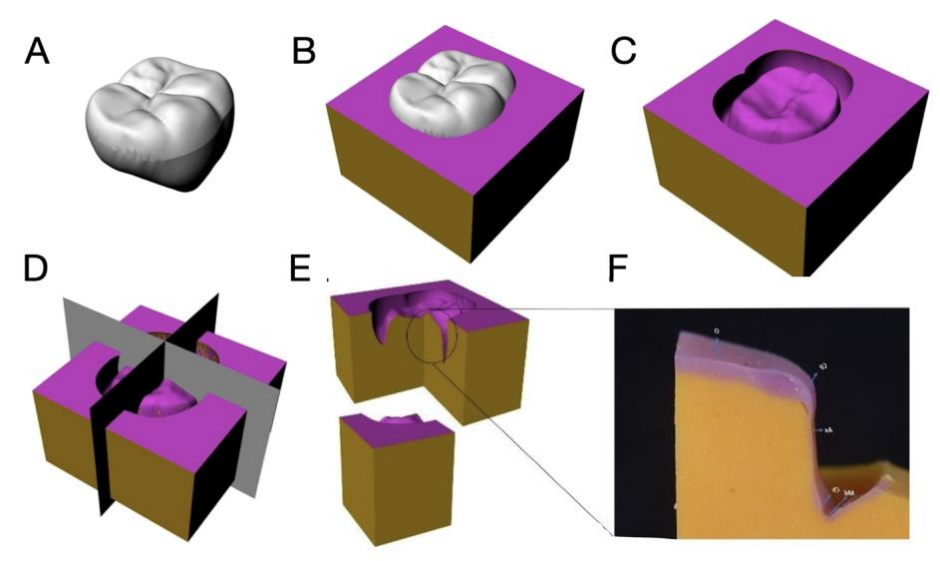
Illustration of the sectioning process of the silicone troquel. A, Representative zirconia crown. B, Silicon troquel manufactured using fluid and putty consistencies. C, Final silicon troquel. D, Bucco-lingual and mesio-distal sections. E, Sectioned silicone troquel in four pieces, totalling eight hemi-faces. F, In vitro analysis of one representative hemi-face with all evaluated regions: occlusal (o), cusp (cp), axial (ax), and chamfer (ch) for internal discrepancy, and the chamfer ending region (finish line) for marginal discrepancy.

With the aid of a scalpel blade Nº. 15C, the silicone replicas were sectioned in two axial directions: one in the mesiodistal direction and another in the vestibulolingual direction, producing 4 fragments (Figure 2A-F), totalling 8 hemifaces. Each slice was subdivided into 4 regions: occlusal (o), cusp (cp), axial (ax), and chamfer (ch) (Figure 2). Therefore, to measure the ID, the thickness of the fluid silicone was measured 5 times in all four regions (o, cp, ax, and ch), totalling 80 measurements per crown. For the measurement of MD, the thickness of the fluid silicone was measured in the chamfer ending region (finish line), with 5 measurements performed on each hemiface, totalling 20 measurements per crown. All measurements were taken in the optical stereomicroscope (Stereo Discovery V20, Zeiss, Göttingen, Germany). Values recorded in micrometers (μm) using software (NIS-Elements D420.00 64-bit). Two-dimensional images were obtained with the Infinity Capture program (Lumenare Corporation, Ottawa, Canada), allowing the measurement of the cement layer and the vertical measurement of the cervical margin of the crown all the way to the surface of the preparation. All cuts and measurements were performed by the same operator, who was unaware of the die material being evaluated.

### Statistical analysis

The sample size was calculated using OpenEpi (Version 3.01) software to compare two means. The input parameters for the calculation were as follows: a two-sided confidence interval of 95%, a power of 80%, and a sample size ratio of 1 (Group 2/Group 1). The means and standard deviations for Group 1 (the control group) and Group 2 (the experimental group) were based on a previous study conducted by our research group ^24^. Group 1 had a mean of 61.6±11.5. While Group 2 had a mean of 78.4±6.

Using these parameters, the required sample size was determined to be eight specimens per group. However, to enhance the reliability and robustness of the study, it was decided to include 10 specimens per group. This calculation ensured sufficient statistical power to detect a clinically significant difference between the groups.

The 100% statistical power calculation was performed on the www.openepi.com site, considering a 95% confidence interval for ID and MD values, separately. After performing the Shapiro-Wilk normality test, the data were found to have a normal distribution. Then, mean and standard deviation values were calculated using a statistical software program (Analytical Software Inc., version 8.0, 2003, Tallahassee, FL/USA). ID data were subjected to a 2-way ANOVA followed by a Tukey test (at the 5% significance level), while MD data were analysed using a 1-way ANOVA. The ANOVAs were performed with 95% confidence intervals (CIs)

## Results

For ID, two-way ANOVA revealed that the factors “material” (p = 0.004), “region” (p = 0.00), and the interaction of factors (p = 0.0258) were significant. When only the factor “ceramic” was evaluated, the highest ID was observed for PIC (148.37^A^), followed by Zr (124.14^B^), and DL (119.20^B^). Similarly, when the factor “region" was evaluated in isolation, the highest results were observed for O (178.65 μm), followed by CP (139.34 μm), which was similar to CH (113.39 μm). Conversely, the lowest result was found for AX (90.91^C^ μm). When the experimental groups were compared among themselves, the groups PICo (226.0±84.7^A^ μm) and PICc (165.6±71.9^AB^ μm) presented the highest values of ID, while the groups LDax (87.9±17.3^D^ μm) and PICax (91.4±36.2^D^ μm) presented the lowest. Additional results and a comparison between the groups are presented in Table 2.

For MD, 1-way ANOVA revealed that the factor “material” was not significant (p = 0.4287). The Zr (108.4 ± 34.6 μm), LD (95.7 ± 13.6 μm), and PIC (114.5 ± 42.0 μm) ceramics exhibited similar MD values (Table 2).

**Table 2:** Mean, standard deviation (μm) for Internal Discrepancy (ID) and Marginal Discrepancy (MD), for lithium disilicate, zirconia, and polymer-infiltrated ceramic crowns (Tukey, 5%)

Group	Material	Region	ID	MD
PICo	Polymer infiltrated ceramic (PIC)	Occlusal	226.0±84.7^A^	114.5±42.0
PICcp		Cusp	165.6±71.9^AB^	
PICax		Axial	91.4±36.2^D^	
PICch		Chamfer	110.35±36.9 ^BCD^	
ZRo	Zirconia (Zr)	Occlusal	155.31±29.8^B^	108.4±34.6
ZRcp		Cusp	132.57±29.4^BCD^	
ZRax		Axial	93.34±20.4^CD^	
ZRch		Chamfer	115.32±28.6^BCD^	
LDo	Lithium disilicate (LD)	Occlusal	154.6±33.7^BC^	95.7 ±13.6
LDcp		Cusp	119.7±21.3^BCD^	
LDax		Axial	87.9±17.3^D^	
LDch		Chamfer	114.5±16.5^BCD^	

*Different letters represent a significant difference for ID (P<0.05).

## Discussion

This in vivo investigation evaluated the ID and MD of Zr, LD, and PIC crowns. Various methods have been described for assessing the internal and marginal discrepancies of indirect restorations in in vivo studies. Among them, the replica technique is considered one of the most suitable, as it is reliable, nondestructive, and easily reproducible.^24^ This technique allows measurements not only on the internal walls but also at the margins.^26^ Several studies have evaluated ID and MD in indirect restorations; however, there is no consensus regarding the specific regions measured internally or the number of measurement points required. For instance, Morys et al. (2021) ^26^ measured three internal points and one marginal point, each with three readings, whereas Rau et al. (2018) ^27^ assessed only MD and occlusal ID, with eight readings per measurement. However, Groten et al (2000) ^(^ ^28^ suggested that at least 50 measurement points are necessary for clinically relevant results.

For this study, the methodology of Zeltner et al. ^29^was applied. The defined measurement regions included the occlusal, axial wall of the preparation, and cusp, with each region measured at five points, spaced approximately 250 μm apart. Additionally, the chamfer region was included to enhance the reliability of internal discrepancy (ID) measurements. For marginal discrepancy (MD), five points were also evaluated, spaced at the same 250 μm distance along the finish line region.^29^ A major challenge in comparing studies is the lack of standardization in ID and MD assessment. For ID, there is no consensus on which specific areas should be evaluated for diagnosis.^26^ For MD, different preparation finishing lines may influence the adaptation of restorations.^27^ The replica technique, while widely used, has limitations, including the risk of silicone tearing and difficulties in accurately reproducing the finishing region.^26^ Additionally, CAD/CAM materials vary in precision, as differences in manufacturing methods and design software can affect the final fit of prosthetic crowns.^26^

Regarding both evaluated discrepancies, Zr, LD, and PIC crowns showed similar results in MD, supporting the first hypothesis that all crowns would exhibit comparable values. Proper prosthetic adaptation is crucial for the longevity of dental restorations, as it minimizes excessive exposure of the cement layer to the oral environment, thereby reducing the risk of degradation.^30^ The impression technique-whether using addition silicone or digital scanning-plays a key role in MD evaluation. Studies comparing conventional addition silicone impressions with intraoral scans report clinically acceptable MD values for both techniques, ranging from 40.02 μm to 30.91 μm, respectively.^26^ Although Morys et al.^26^reported lower MD values, they were also associated with larger standard deviations. The authors evaluated two different impression techniques-addition silicone and intraoral scanning-but found no significant difference in MD between them. In the present study, additional silicone molds were used to create gypsum models, which were then scanned using laboratory bench scanners, following a process similar to that of Morys et al.^26^

Another study evaluated four CAD/CAM systems for producing monolithic lithium disilicate crowns ^31^, setting the internal gap parameter at 50 μm. The authors observed the highest MD (133.01 μm) in the Ceramill system and the lowest MD (95.2 μm) in the Zirkonzahn system. The present findings align with Rau (2018),^27^ who reported an MD of 104 μm for Zr crowns. Similarly, Cho et al. (2023)^32^investigated MD in different aspects of the preparation (buccal, mesial, distal, and lingual), reporting the lowest MD (81.44 μm) in the buccal aspect and the highest MD (92.82 μm) in the lingual aspect.

The thickness of the cement layer, represented by ID, can significantly influence crown retention and fracture resistance.^33^ Excess cement can disrupt force transmission within the tooth-restoration complex, compromise crown support, and potentially lead to restoration displacement and premature fracture.^19^ Several factors can affect the fit accuracy of ceramic crowns, including the CAD/CAM milling system, measurement method, number and location of measurement points, restorative material type, and preparation design.^34^ According to the American Dental Association (ADA), the ideal cement thickness should range from 25 to 40 μm.^35^ However, achieving this in clinical practice is challenging. As a result, cement thicknesses of up to 120 μm and 150 μm have been deemed clinically acceptable.^28^ Iwai et al. (2008)^36^ reported that the predefined cement space also influences the marginal fit of zirconia crowns, as cement volume loss can occur. A cement space smaller than 60 μm may cause unintended interface contacts, preventing proper crown seating, impeding excess cement flow, and ultimately increasing MD.^31^

The second hypothesis was accepted, as ID values varied across evaluated regions. The advancement of digital workflow systems has improved ID accuracy.^34^ However, studies analyzing indirect scanning of silicone preparation replicas indicate that scanning efficiency decreases in non-flat areas.^37^ This explains the higher ID values observed in occlusal (o), cusp (cp), and chamfer (ch) regions, where cement material tends to accumulate. Additionally, more anatomical preparations may result in higher ID values.^38^ Preparation surface variations can also affect ID,^39^ as rounded areas are more difficult to mill. The occlusal region typically shows greater irregularities, as milling in this area is performed by the tip of the bur, which is disproportionate to the restoration size, resulting in lower definition.^40^ In contrast, the axial (ax) region, which is milled with the side of the drill, tends to have greater accuracy.^34^ Morys et al. (2021)^26^reported that higher occlusal ID values may be related to CAD software manipulation of virtual models, where peaks and valleys in occlusal and incisal areas are automatically rounded-a phenomenon known as "overshooters”.

The literature on MD and ID of monolithic zirconia crowns remains limited. Rau et al. (2018)^37^ clinically evaluated MD and ID exclusively in the occlusal region, reporting clinically acceptable MD (104 μm) and ID (101 ± 41 μm). However, other internal regions were not assessed in their study. Moreover, the restorative material significantly influenced ID, which can be attributed to the different post-milling processes that were employed. LD is milled in a pre-sintered stage and then subjected to a crystallization cycle in a furnace at 840°-850°C for 10 minutes. Zr, on the other hand, undergoes sintering at 1500°C after milling, leading to material shrinkage of up to 30%.^1^ In contrast, PIC does not require a post-milling firing process and is ready for use immediately.^10^

Given that the results of this study align with those reported in vitro ^36^, long-term clinical follow-up is necessary to confirm these in vivo findings on ID and MD and to assess their impact on the longevity of restorative treatment. Accordingly, this study will continue with long-term follow-up.

## Conclusion

Based on the findings of this study, ultratranslucent zirconia, lithium disilicate, and polymer-infiltrated ceramic crowns exhibited similar performance, with clinically acceptable internal and marginal discrepancy values. Notably, the highest internal discrepancy was observed in the occlusal region.
